# The Structural Imaging Characteristics and Its Clinical Relevance in Patients with Cerebral Venous Thrombosis—A Retrospective Analysis from One Single Center in China

**DOI:** 10.3389/fneur.2017.00648

**Published:** 2017-11-30

**Authors:** Li-xin Zhou, Ming Yao, Li-ying Cui, Ming-li Li, Yi-cheng Zhu, Jun Ni, Bin Peng

**Affiliations:** ^1^Department of Neurology, Peking Union Medical College Hospital, Chinese Academy of Medical Science, Beijing, China; ^2^Neuroscience Center, Chinese Academy of Medical Sciences, Beijing, China; ^3^Department of Radiology, Peking Union Medical College Hospital, Chinese Academy of Medical Science, Beijing, China

**Keywords:** cerebral venous thrombosis, imaging, parenchymal lesion, intraluminal thrombosis, magnetic resonance imaging

## Abstract

**Background:**

Few studies have investigated structural imaging findings of cerebral venous thrombosis (CVT) in China. The structural imaging [head computed tomography (CT) and routine brain magnetic resonance imaging (MRI)] can assess any parenchymal lesion secondary to the venous thrombosis and reveal direct signs of intraluminal thrombus. In recent years, many patients can be diagnosed with CVT more rapidly and directly by structural imaging. The aim of the present study is to determine the performance of structural imaging in the diagnosis and outcome of CVT in a large cohort single center of Chinese patients.

**Methods:**

We evaluated consecutive patients admitted to our hospital with CVT receiving structural imaging from 1991 to 2015. A neuroradiologist, blinded to clinical data, independently reviewed the structural imaging, including head CT and routine MRI for parenchymal lesions and signs of dural venous sinus thrombosis, as well as the MRV/DSA findings. The Clinical and laboratory data were reviewed and recorded for further analysis.

**Results:**

117 patients were included in this study, 68 (58.1%) were females. Parenchymal lesions were identified in 56.4% (66/117) of the patients on structural imaging, including focal edema in 30.8%, hemorrhage in 19.7%, and brain swelling in 4.3% of the patients. Patients with parenchymal lesions presented with more often seizures (*P* < 0.001) and less often headache (*P* = 0.049). Intraluminal thrombus within the sinuses or veins on structural imaging was found in 28.2% (33/117) of the patients. Patients with both intraluminal thrombus and parenchymal lesions on structural imaging had more acute onset (*P* = 0.01) and present more consciousness disturbance (*P* = 0.007).

**Conclusion:**

Intracranial lesions on structural imaging are frequently found in patients with CVT. Patients with parenchymal lesions on structural imaging, especially with intraluminal thrombus simultaneously, tend to have a severe clinical picture and might lead to a devastating or fatal outcome. Structural imaging may help on early diagnosis and predict the poor outcome of CVT.

## Introduction

Cerebral venous thrombosis (CVT) is an uncommon but potentially serious and life-threatening cause of stroke, accounting for 0.5–1% of all strokes (1). Since the underlying risk factors and clinical manifestations of CVT are highly diverse which make early diagnosis much difficult, neuroimaging plays a key role in the diagnosis and further treatment (2).

The structural imaging [head computed tomography (CT) and routine brain magnetic resonance imaging (MRI)] can assess any parenchymal lesion secondary to the venous thrombosis and reveal direct signs of intraluminal thrombus (3). As some special MRI sequences, such as susceptibility-weighted images, are used in routine MRI protocols to detect the presence of intraluminal thrombus, many patients can be diagnosed with CVT more rapidly and directly by structural imaging in recent years (4, 5). On the other hand, although the parenchymal lesions are not specific, they could draw attention and prompt a search for direct visualization of a thrombus on MRI or ordering a venography. Therefore, understanding the characteristics of structural imaging for the evaluation of CVT is especially important when CVT is not clinically suspected or magnetic resonance venography (MRV) has not been ordered. In addition, systematic studies investigating structural imaging characteristics in CVT are scarce from China. The aim of the present study is to determine the structural imaging performance on the diagnosis and outcome of CVT in a large number of Chinese patients from a single center.

## Materials and Methods

### Patients

**Figure 1:**
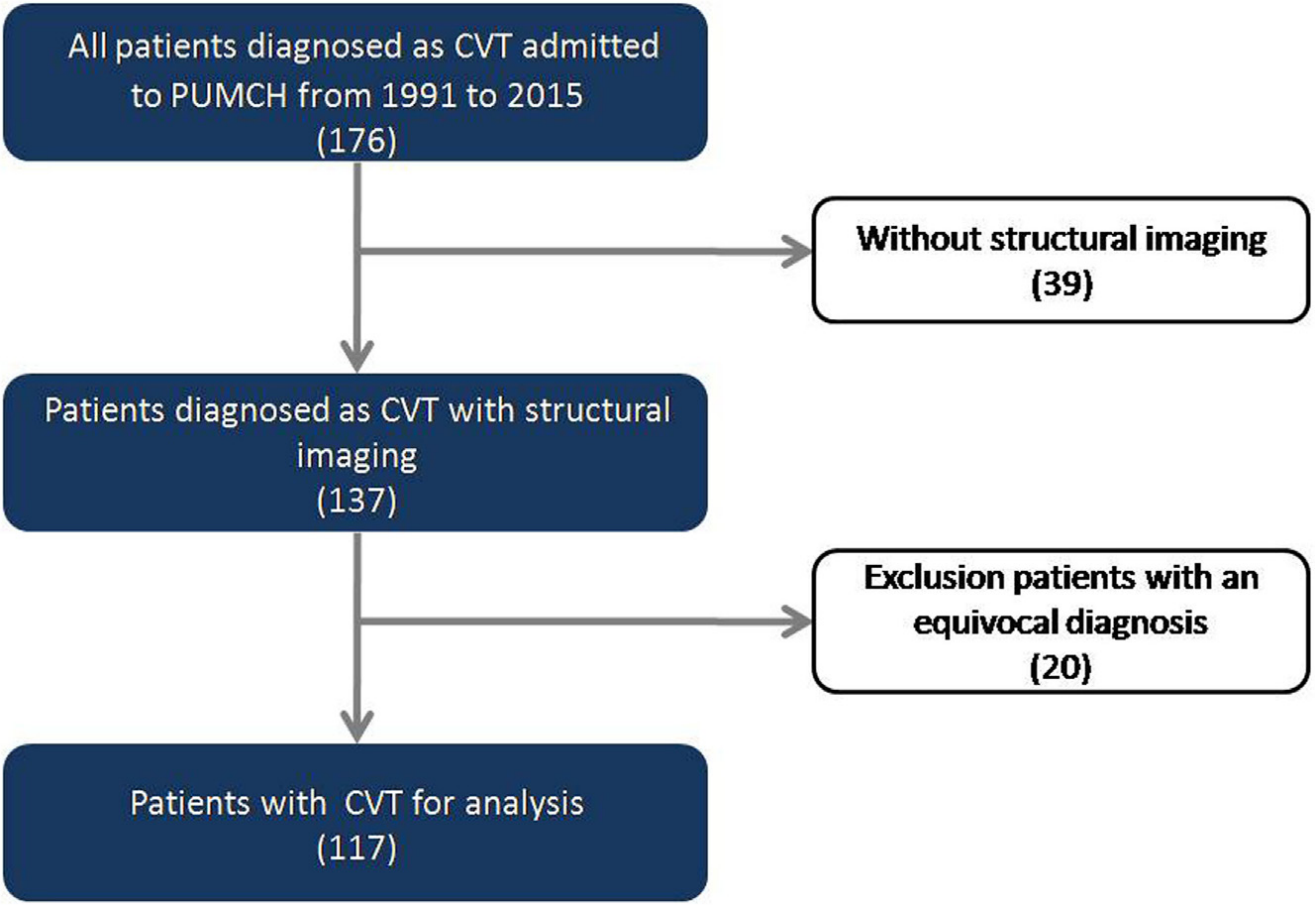
Flowchart of the screening process for identifying patients with cerebral venous thrombosis (CVT).

This was a single-center retrospective hospital-based study. We reviewed our stroke database of all consecutive patients diagnosed as CVT admitted to Peking Union Medical College Hospital between July 1991 and December 2015. All of the patients enrolled in this study had at least one of the following neuroradiological studies to confirm the diagnosis of CVT: CT scan, MRI, MRV, or cerebral angiography, following established diagnostic criteria (3). Of 176 consecutive patients with a diagnosis of CVT,39 without available head CT or brain MRI or with low-quality images were excluded. Given some patients before 2000 might be misdiagnosed due to the limited imaging information, all clinical and neuroimaging records were reviewed by our cerebrovascular group and reconfirmed the diagnosis of CVT. Based on the group review, 20 patients with an equivocal diagnosis were further excluded, leaving 117 patients for analysis in the present study (Figure 1). The ethics committee of Peking Union Medical College Hospital approved the protocol of the study.

### Baseline Data

We reviewed medical records extensively and recorded demographic data, clinical presentations, risk factors such as genetic and acquired prothrombotic states, trauma, and infections or inflammatory conditions, pregnancy and puerperium, laboratory testing, treatments, and outcome on discharge.

### Imaging Protocol

The MRI protocol used in the study routinely includes T1-weighted, T2- weighted, diffusion-weighted images (DWI), non-echo-planar fluid-attenuated inversion recovery (FLAIR), and MRV or cerebral angiography. T2*-weighted gradient echo (GRE) was conducted in some of the patients after 2000. The admission imaging was used for analysis. The initial imaging performed after symptom onset was used in the study if the patients were not admitted to our hospital primarily. Of 117 patients enrolled in the present study, 89 performed MRV and 52 performed cerebral angiographies.

In this study, the parenchymal lesions secondary to CVT on structural imaging were categorized as three subtypes: brain swelling, focal edema, and hemorrhage [parenchymal hemorrhage or subarachnoid hemorrhage (SAH)] (6). Brain swelling was defined as there were no abnormalities in attenuation or signal intensity on structural images, but sulcal effacement, diminished cistern visibility, and a reduction in ventricular size may occur. Focal edema is visible on CT or MR images by focal abnormalities in attenuation or signal intensity without visible hemorrhage, which could be subclassified as either vasogenic edema (with increased ADC values presumably related to venous congestion) or cytotoxic edema (with decreased ADC values related to cellular energy disruption) by DWI techniques. The definition of intraluminal thrombus was the cord sign and the dense sign on plain CT or the empty delta sign on enhance CT scan or abnormal high signal on T1, loss of flow void on T2, loss of flow void or hyperintense signal on FLAIR in the corresponding venous sinus and veins, accompanied by filling defect. In each case, evaluation of all sequences of interest was performed at the same time to reflect usual clinical practice.

A radiologist blinding to clinical information rated all images, including head CT, MRI, MRV, and digital subtraction angiography. The supervisor will further review and confirm the lesions, which were originally doubted.

### Statistical Analysis

The continuous demographic data were analyzed using mean ± SD and the discrete data using number and percentage. Differences in proportions were tested with chi-square test or Fisher’s exact test when required. Data analysis was processed on SPSS Statistics Bass 20.0 (SPSS Inc., Chicago, IL, USA). Student’s *t*-test was used for continuous variables. All *P* values were two-tailed and criteria for significance were *P* < 0.05.

## Results

### Baseline Clinical Data

The mean age of patients diagnosed with CVT was 34 ± 14.3 years. There was a female predominance (58.1%, 68/117). The onset of CVT in the study was almost acute or subacute (within 2 weeks) in 89.7% (105/117) of the cases. The most common manifestation was headache (87.2%, 102/117), followed by seizures (31.6%, 37/117), focal neurological deficits (29.9%, 35/117), visual impairment (26.5%, 31/117), and consciousness disturbance (15.4%, 18/117). At least one underlying risk factor was identified in 71.8% (84/117) of patients. By location, the common affected sinuses were transverse sinus (65.0%, 76/117), sigmoid sinus (55.6%, 65/117), and superior sagittal sinus (54.7%, 64/117). Only 4.3% (5/117) of patients had isolate thrombosis within the cortical veins.

In hospitalization, most of the patients (82.1%, 96/117) received anticoagulation with intravenous heparin or subcutaneous low-molecular weight heparin (LMWH) in therapeutic dosages. Data of clinical outcome at discharge were available for 86.3% (101/117) of the patients. A total recovery (41.2%) or clinical improvements (50.5%) were found in majority of the cases (92.1%, 93/101). In-hospital mortality was 4.0% (4/101) in our cohort.

### Parenchymal Lesions on Structural Imaging

**Figure 2:**
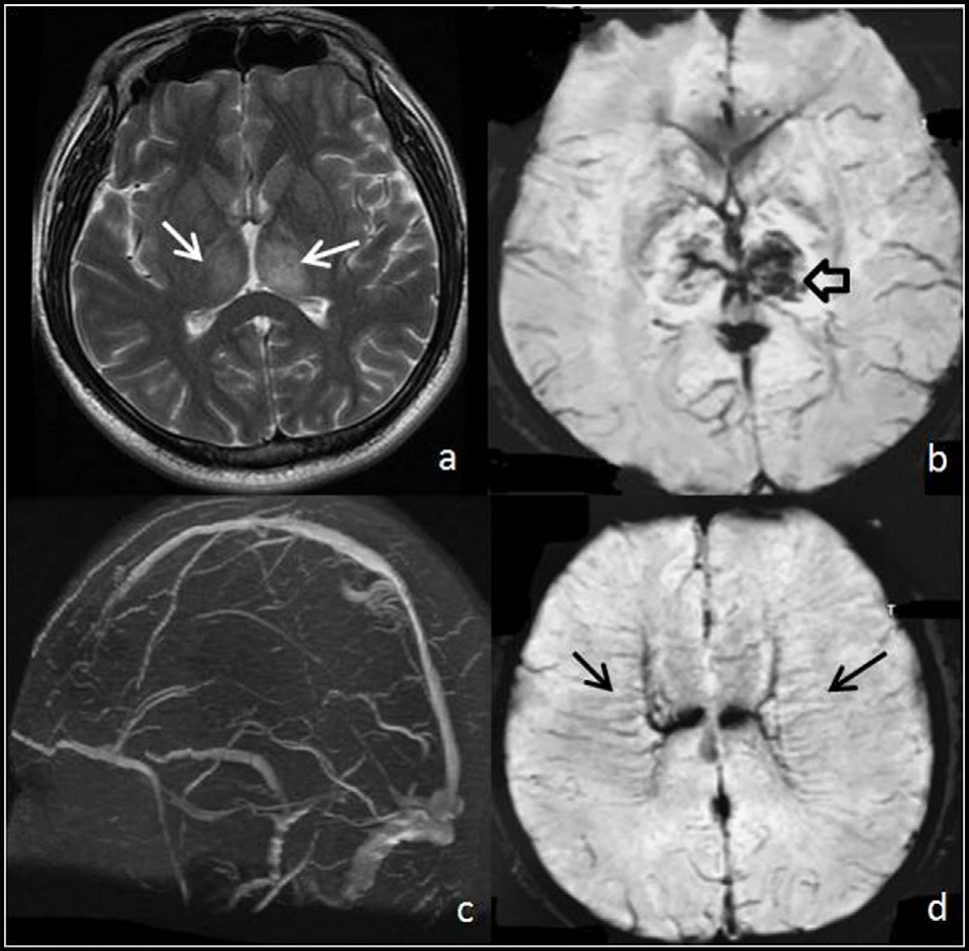
Thrombosis of deep venous system. **(A,B)** T2 and T2* images show focal edema (white arrows) and hemorrhage (hollow arrow) within both thalami. **(C)** MR venogram demonstrates a portion of the deep venous system with no signal, a finding consistent with occlusion. **(D)** T2* images show multiple linear abnormal signals within the deep white matter (black arrows), which demonstrate medullar veins dilated compensatory.

Parenchymal lesions were identified on structural imaging in 56.4% of the patients (66/117). Focal edema was the most common structural imaging findings, which was visible on CT and MRI in 30.8% (36/117) of the patients. Hemorrhage was detected in 19.7% (23/117) of cases, including parenchymal hemorrhage in 15.4% (18/117) and SAH in 4.3% (5/117) of the cases. Brain swelling was visualized on CT and MRI in only 4.3% (5/117) of cases. In two patients, multiple dilated subcortical and medullar veins were observed on T2*-GRE (Figure 2).

Compared to the patients without parenchymal lesions, those with parenchymal lesions presented with more often seizures (*P* < 0.001) and less often headache (*P* = 0.049). More patients with parenchymal lesions were anticoagulated than those without parenchymal lesions (*P* = 0.019). There was no significant difference in demographic data, onset of the CVT, occluded sinus and veins, underlining risk factors, and outcome at discharge between two groups. The comparison of baseline data, clinical presentation, risk factors, and occluded sinus or vein between patients with and without parenchymal lesions is presented in Table 1.

**Table 1:** The comparison of baseline data, clinical presentation, risk factors and radiological findings of patients with and without parenchymal lesions.

	Patients with parenchymal lesions (*n* = 66)	Patients without parenchymal lesions (*n* = 51)	*P* value
Demographic data			
Age (mean ± SD)	36 ± 13.9	32 ± 14.6	0.054
Female sex	42 (62.6%)	26 (51.0%)	0.171
Onset of cerebral venous thrombosis			0.617
Acute	22 (33.3%)	14 (27.5%)
Subacute	37 (56.1%)	32 (62.7%)
Chronic	7 (10.6%)	5 (9.8%)
Clinical presentation			
Headache	54 (81.8%)	48 (94.1%)	0.049
Visual impairment	15 (22.7%)	16 (31.4%)	0.295
Focal neurological deficits	24 (36.4%)	11 (21.6%)	0.084
Seizure	28 (42.4%)	9 (17.6%)	<0.001
Consciousness disturbance	11 (16.7%)	7 (13.7%)	0.663
Risk factors			0.827
Prothrombotic conditions	12 (18.2%)	11 (21.6%)
Autoimmune system disease	8 (12.6%)	8 (15.7%)
Infection	9 (13.6%)	5 (9.8%)
Hematologic disorders	7 (10.6%)	7 (13.7%)
Pregnancy and puerperium	8 (12.6%)	0
Drugs	2 (3.0%)	1 (2.0%)
Tumor	4 (6.1%)	1 (2.0%)
Trauma	1 (1.5%)	0
Unknown	15 (22.7%)	18 (35.3%)
Occluded sinus or vein			
Superior sagittal sinus	37 (56.1%)	27 (52.9%)	0.670
Inferior sagittal sinus	8 (12.1%)	3 (5.9%)	0.243
Transverse sinus	43 (65.2%)	33 (64.7%)	0.871
Sigmoid sinus	36 (54.5%)	29 (56.5%)	0.874
Straight sinus	13 (19.7%)	5 (9.8%)	0.136
Deep system	7 (10.6%)	2 (3.9%)	0.173
Cortical vein	3 (4.5%)	2 (3.9%)	0.859
Anticoagulation treatment	59 (89.4%)	37 (72.5%)	0.019
Outcome at discharge			0.249
Complete recovery	19 (32.8%)	23 (53.5%)
Improvement	34 (58.6%)	17 (39.5%)
Unchanged	1 (1.7%)	2 (4.7%)
Worsen	0	1 (2.3%)
Death	4 (6.9%)	0
Unknown	8	8

### Intraluminal Thrombus in Sinuses or Veins on Structural Imaging

**Figure 3:**
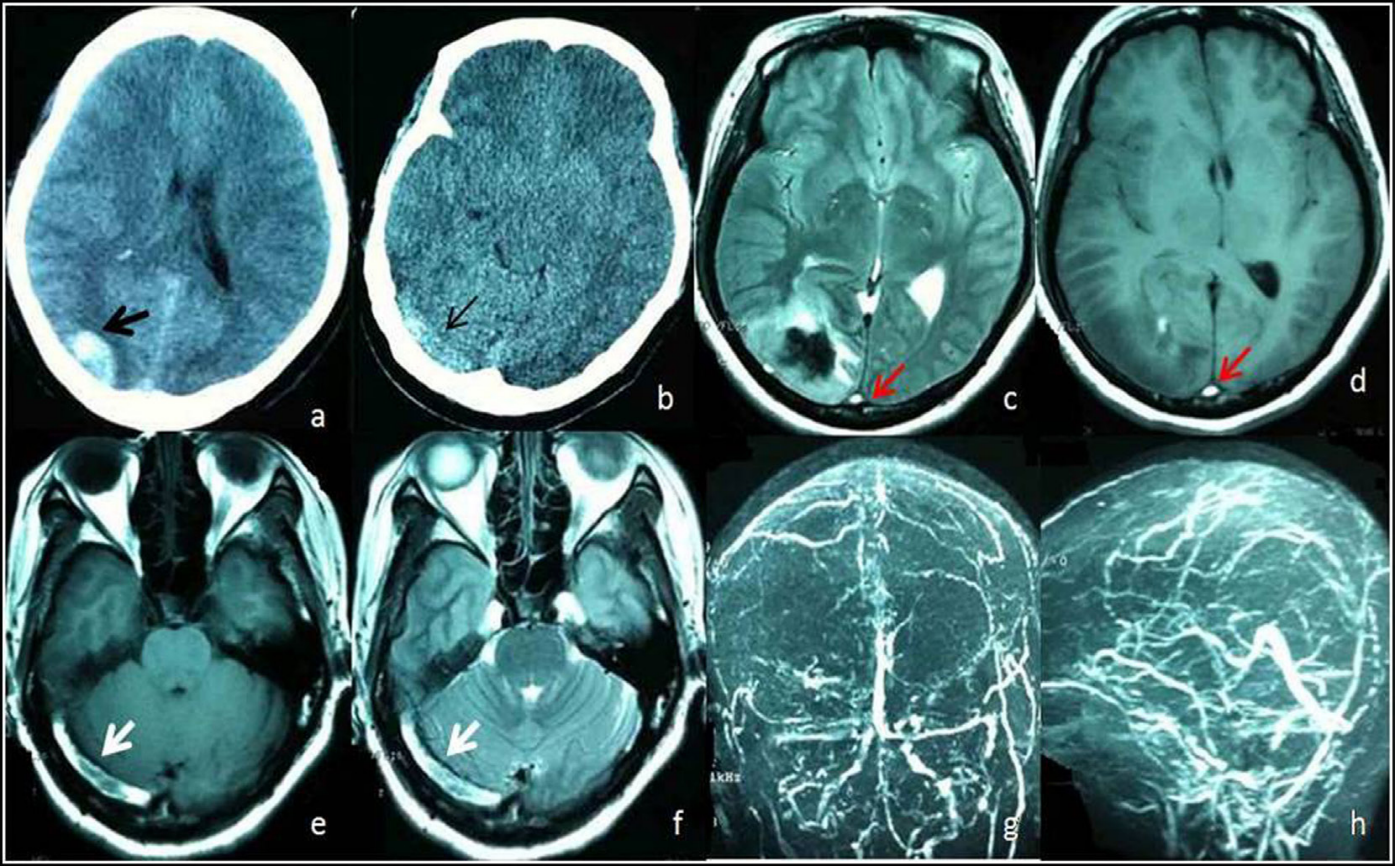
A 48-year female admitted to Emergency Room with headache and vomiting. **(A)** Head computed tomography (CT) shows hemorrhage on right occipital lobe (black arrow). **(B)** Unenhanced CT image shows areas of abnormal hyperattenuation consistent with thrombus in right transverse sinuses (thin arrow). Axial T1-weighted **(D,E)** and axial T2-weighted **(C,F)** MR images acquired 10 days after onset show an area of abnormal increased signal intensity in the superior sagittal sinus (red arrow) and right transverse sinus (white arrow) consistent with subacute thrombi of the superior sagittal sinus and right transverse sinus. Magnetic resonance venography **(G,H)** shows occlusion of the superior sagittal sinus and filling defects within the right transverse sinus (hollow arrow) due to thrombi.

Intraluminal thrombus within the sinuses or veins was found on structural imaging in 28.2% (33/117) of the patients (Figure 3). Of these, intraluminal thrombus was detected by head CT in 6.0% (7/117), by brain MRI in 20.5% (24/117), and by both CT and MRI in 1.7% (2/117) based on the definition.

In the present study, 13 patients have both parenchymal lesions and intraluminal thrombus on structural imaging. Compared to the patients (*n* = 31) with neither parenchymal lesions nor intraluminal thrombus, they had significantly more acute onset (*P* = 0.01) and presented more consciousness disturbance (*P* = 0.007). In-hospital mortality was higher (15.3% vs 0%).

## Discussion

The present study is the first report of the structural imaging findings in a large Chinese CVT cohort. In the present series, the female predominance, age of onset, frequency of onset, and varied clinical manifestations are those classically reported in previous large series of CVT (7). Our finding on mortality at discharge was quite similar to the result of the International Study on Cerebral Vein and Dural Sinus Thrombosis (ISCVT), in which mortality at discharge was only 4.3% (7).

### Parenchymal Lesions on Structural Imaging

Parenchymal lesions were observed in 56.4% of a total of 117 patients on structural imaging, in line with the previous report (8). However, brain swelling was detected in only 4.3% of the patients in our cohort. Previous studies have reported that brain swelling may occur in as many as 42% of patients with CVT (9). A single center design and the ethnic difference might partly explain the relative low percentage of brain swelling observed in our cohort. An alternative explanation is that potential different parameters among patients would affect the diagnosis even all the imaging were re-evaluated. Finally, the duration between symptoms onset and performance of imaging might affect the visibility of brain swelling. Patients presented with only brain swelling but without parenchymal signal intensity changes tend to have intrasinus pressures in the intermediate range (20–25 mmHg); however, intrasinus pressures could also be markedly elevated (10). Such patients typically have more prominent clinical symptoms than expected based on the imaging findings. Thus, we suggest clinicians and radiologists to promptly identify the brain swelling in the patients with CVT.

In addition, we found that seizures occurred more frequently in patients with parenchymal lesions. Our finding was in line with that reported from the ISCVT, showing that 245 (39%) of 624 patients presented with seizures (11). The findings demonstrated that patients with parenchymal lesions have a higher risk of seizures and supported the prescription of antiepileptic agents in CVT patients with parenchymal lesions who present with the first attack of seizure. However, for those patients without seizure attack but with parenchymal lesions, whether the anticonvulsants should be used or not still remains controversial (11, 12). Further large-sample randomized studies are warrant to investigate the safety and effect of the prophylactic use of antiepileptic drugs on CVT patients with parenchymal lesions but without seizure attack.

### Intraluminal Thrombus in Sinuses or Veins on Structural Imaging

In our cohort, only 28.2% (33/117) of the patients were detected with the direct signs of intraluminal thrombus in sinuses or veins on structural imaging. A few recent studies demonstrated that the combination of routine MR sequences had an overall sensitivity of 79%–83% on detecting intraluminal thrombus (5, 13). The lower prevalence of intraluminal thrombus on structural imaging in our study might be related to the variable interval between the onset of thrombus formation and the time of imaging. As we know, the magnetic resonance signal intensity of venous thrombus varies according to the time of imaging from the onset of thrombus formation. In addition, recognition of these direct signs might be challenged due to less knowledge of the different stages of thrombus evolution and pitfalls. Another reason might be that only a small part of patients in our center performed GRE sequences. As GRE sequences are increasingly used to detect the presence of blood breakdown products, their usefulness in depicting intraluminal thrombosis in CVT has been appreciated (14, 15).

In our study, patients with both intraluminal thrombus and parenchymal lesions on structural imaging had significantly more frequent seizures with consciousness disturbance and high percentage of in-hospital mortality, which suggest that these patients have a severe clinical picture and might lead to a devastating or fatal outcome. Thus, the clinicians should be highly alert the early signs of intraluminal thrombus on structural imaging and give the patients early diagnosis and prompt treatment in order to prevent the unfavorable outcome.

Our study had some limitations. First, the retrospective nature of the study resulted in a non-uniform method of neuroimaging studies. Second, given the study was carried out with patients from a single referral center, selected bias might have contributed to the inclusion of more acute-onset and severe cases in the study than would otherwise have been the case. Finally, patients were just followed until hospital discharge, and thus, there were inadequate data to compare the long-term clinical outcome.

In conclusion, intracranial lesions on structural imaging are frequently found in patients with CVT in our cohort. Seizures occurred more frequently in patients with parenchymal lesions. Patients with parenchymal lesions and intraluminal thrombus simultaneously on structural imaging tend to have a severe clinical picture and might lead to a devastating or fatal outcome. Structural imaging may be of help on early diagnosis and prediction of the outcome of CVT.

## Author Contributions

L-xZ and JN contributed to the writing of the article. MY and Y-cZ contributed to the collection of data. M-lL contributed to the rating of images. L-yC and BP contributed to the revising of the article.

## Conflict of Interest Statement

The authors declare that the research was conducted in the absence of any commercial or financial relationships that could be construed as a potential conflict of interest.
